# Automated image analysis system for studying cardiotoxicity in human pluripotent stem cell-Derived cardiomyocytes

**DOI:** 10.1186/s12859-020-3466-1

**Published:** 2020-05-14

**Authors:** Lu Cao, Andries D. van der Meer, Fons J. Verbeek, Robert Passier

**Affiliations:** 1 Imaging and Bioinformatics group, Leiden Institute of Advanced Computer Science (LIACS), Leiden University, Niels Bohrweg 1, Leiden, 2333 CA The Netherlands; 2 Dept of Applied Stem Cell Technologies, MIRA Institute, University of Twente, Drienerlolaan 5, Enschede, 7522 NB The Netherlands; 3 Dept of Anatomy and Embryology, Leiden University Medical Center, Albinusdreef 2, Leiden, 2333 ZA The Netherlands

**Keywords:** Cardiotoxicity, hiPSC-derived cardiomyocytes, High-throughput screening, Image analysis, Phenotype quantification

## Abstract

**Background:**

Cardiotoxicity, characterized by severe cardiac dysfunction, is a major problem in patients treated with different classes of anticancer drugs. Development of predictable human-based models and assays for drug screening are crucial for preventing potential drug-induced adverse effects. Current animal in vivo models and cell lines are not always adequate to represent human biology. Alternatively, human induced pluripotent stem cell-derived cardiomyocytes (hiPSC-CMs) show great potential for disease modelling and drug-induced toxicity screenings. Fully automated high-throughput screening of drug toxicity on hiPSC-CMs by fluorescence image analysis is, however, very challenging, due to clustered cell growth patterns and strong intracellular and intercellular variation in the expression of fluorescent markers.

**Results:**

In this paper, we report on the development of a fully automated image analysis system for quantification of cardiotoxic phenotypes from hiPSC-CMs that are treated with various concentrations of anticancer drugs doxorubicin or crizotinib. This high-throughput system relies on single-cell segmentation by nuclear signal extraction, fuzzy C-mean clustering of cardiac *α*-actinin signal, and finally nuclear signal propagation. When compared to manual segmentation, it generates precision and recall scores of 0.81 and 0.93, respectively.

**Conclusions:**

Our results show that our fully automated image analysis system can reliably segment cardiomyocytes even with heterogeneous *α*-actinin signals.

## Background

Cardiotoxic side-effects of anticancer therapy have been known for decades. These adverse effects are found in patients who are treated with anti-cancer drugs from different classes. This includes the earliest chemotherapeutics, i.e. anthracyclines, such as Doxorubicin [1], but also novel therapeutic compounds developed to specific target molecules, such as monoclonal antibodies and small molecules inhibiting tyrosine kinases; e.g. crizotinib [2] and sunitinib [3]. The latter have shown serious cardiotoxicity effects. Since survival of patients who underwent anticancer treatment has improved significantly in the last decade [4], late onsets of adverse cardiovascular effects are more visible in these patients.

Cardiotoxicity is routinely evaluated throughout the drug development process. However, a major reliance on non-human animal models cannot adequately represent human biology. Remarkably, the cardiotoxic effects of the tyrosine kinase inhibitors pazopanib, sunitinib and sorafenib in humans were not detected in animal models [5]. Thus, there is an urgent need for high-content human in vitro systems that can better predict drug-induced toxicity early in the drug development process [6].

The reprogramming of human somatic cells to induced pluripotent stem cells (hiPSC) was described for the first time in 2007 [7]. Now, over a decade later, the differentiation efficiency of hiPSC to specialized cell types, including functional cardiomyocytes, has improved enormously. Moreover, many studies have shown the importance of hiPSC-derived cardiomyocytes (hiPSC-CMs) for disease modelling and drug-induced toxicity screenings [8–10]. Given the fact that toxic side-effects are not shown in every patient, a representation of all significant genetic variants of the population is necessary for detection of cardiotoxicity in the early drug development process, which is exactly what can be achieved by using hiPSC-CMs [11–13].

There are several technologies using fluorescent read-outs to reflect dynamic changes of hiPSC-CMs. These fluorescent read-outs are created by voltage-sensitive or calcium-sensitive dyes [14–16]. The collected fluorescent signals represent electrical and calcium transient signal through time. Phenotypic properties can be extracted from transient signals for further analysis. Although these technologies are fluorescence based and suitable for high throughput, they analyze the average signals from many cells regardless of the celluar heterogeneity in hiPSC-CMs [17]. In order to grasp the dynamics of the cell population, we intend to provide a solution with a focus on single cell analysis by providing phenotypic output, e.g. cell number/morphological features of single cell using high throughput image analysis techniques.

High-throughput image analysis has been shown to be a valuable method for identification of different molecules and drugs that interfere with biological signaling pathways or related functional responses using different cell sources and models. Recently, high-throughput image analysis has also been applied on cardiomyocytes, for example in identifying phenotype changes in *α*-actinin-labeled primary cardiomyocytes from newborn rats [1]. High-throughput imaging has also been applied in studying cardiotoxicity in hiPSC-CMs [18, 19]. However, the high-throughput image analysis in all studies using hiPSC-CMs involved manual steps, mainly to set thresholds for positive signals [20] or for segmentation of individual nuclei regions using commercial image analysis systems [18, 19]. This means that current high-throughput analysis of cardiotoxicity in hiPSC-CMs are not suitable for performing fully automated analysis on the single cell level. The main reason for this lack of full automation is that there are currently no reported robust and automated segmentation methods for high-throughput image analysis of *α*-actinin stained hiPSC-CMs that can subsequently be applied in cytotoxicity studies based on single cell level.

Here we report a fully automated and robust image analysis system, designed for quantification of cardiotoxic phenotypes as measured in datasets obtained from immunofluorescence imaging of *α*-actinin in hiPSC-CMs in a high-throughput setting. We apply an accelerated Fuzzy c-mean clustering algorithm, automatically taking into consideration signal heterogeneity of hiPSC-CMs in terms of size and *α*-actinin signal. We demonstrate proof-of-concept by showing cardiotoxic effects of doxorubicin and crizotinib, which shows a good correlation with manual scoring.

## Methods

### Cell culture

hiPSC-CMs (Pluriomics BV, The Netherlands), obtained at day 14 of differentiation, were thawed and seeded in a Corning 96 well special optics plate (Sigma-Aldrich), coated with Matrigel (40 *μ*g/ml) on day 0. The cell density was 10,000 for each well. The chosen density level is based on our previous testing experiment. At a lower density (5000 cells per well), the cells are not happy and will affect the result. At higher densities (15,000 and 20,000 cells per well), there are too many clumpy cells which increase the difficulty of analysis. The cells were maintained in a humidified incubator at 37 ^∘^C and 5% CO_2_ for 24 hours. Cells were then refreshed with Cardiomyocyte medium (Pluriomics BV) every other day. The composition of the medium is described in the paper [21]. The cells were used for treatment with anticancer drugs on day 9 after seeding.

### Cytotoxicity

To assess the phenotype changes following exposure to drugs, hiPSC-CMs were treated with dimethylsulfoxide (DMSO 4.23 mM) as control or with 0.1 *μ*M, 0.3 *μ*M, 1 *μ*M, 3 *μ*M and 10 *μ*M doxorubicin or crizotinib for 4 days and fixed in 2% paraformaldehyde for 30 minutes at room temperature. A dose range between 0.1 *μ*M and 10 *μ*M was used since it is relevant to the clinical use [18]. An antibody against *α*-actinin, an actin-binding protein that is localized at the Z-disc, of sarcomeres, was used to identify cardiomyocytes, displaying its specific striated pattern [18]. This cardiomyocyte protein is important for evaluating phenotypic maturity of hiPSC-CMs [22] and it provides additional information on cell morphology. Cells were permeabilized using 0.1% Triton X-100 in Dulbecco’s phosphate-buffered saline (DPBS) and incubated with primary antibody in DPBS and 4% goat serum at room temperature for 1 hour. After washing with 0.05% Tween-20 three times, cells were incubated with the secondary antibody in DPBS and 4% goat serum at room temperature for 1 hour. Cells were washed with 0.05% Tween20 again for three times and washed once with DPBS for 5 minutes. For nuclei detection, cells were incubated with DAPI in DPBS (1:1000) for 5 minutes in room temperature and washed three times shortly with DPBS.

### Imaging

**Fig. 1:**
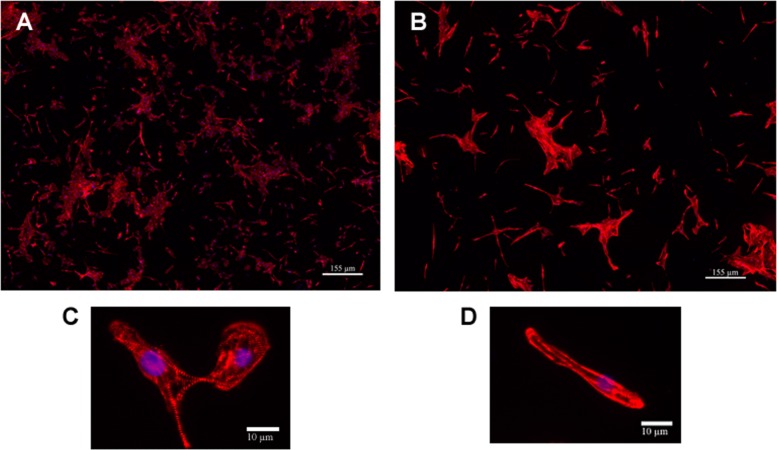
Sample images acquired from BD pathway 855 microscope with 7x7 montage setup. **a** Sample image in the control condition (DMSO). **b** Sample image with 1 *μ*M doxorubicin treatment. **c** A close-up sample image from control condition (DMSO). **d** A close-up sample image with 1 *μ*M doxorubicin treatment

Images of cardiomyocytes were acquired using a high-throughput, high-content imaging BD pathway 855 microscope equipped with a 20x LWD Olympus objective (NA 0.75) and a Hamamatsu - ORCA AG CCD camera. The red-channel (*α*-actinin) was acquired using HQ548/20 excitation filter with 0.08 s exposure time plus 2 gains and acquired a 84101 (84101m, from Chroma 84000 Set) emission filter. The blue-channel was used for the cell nuclei. The signal of DAPI stained cell nuclei was acquired using 380/10-nm excitation filter with 0.0078 s exposure time and the 435LP (Chroma filter) emission filter. The whole monolayer cell culture was scanned through 7x7 (width x height) adjacent image tiles that were stitched to one montage image of 4700x3600 pixels. In Fig. 1, several sample images with different treatment conditions are shown.

### Image analysis

**Fig. 2:**
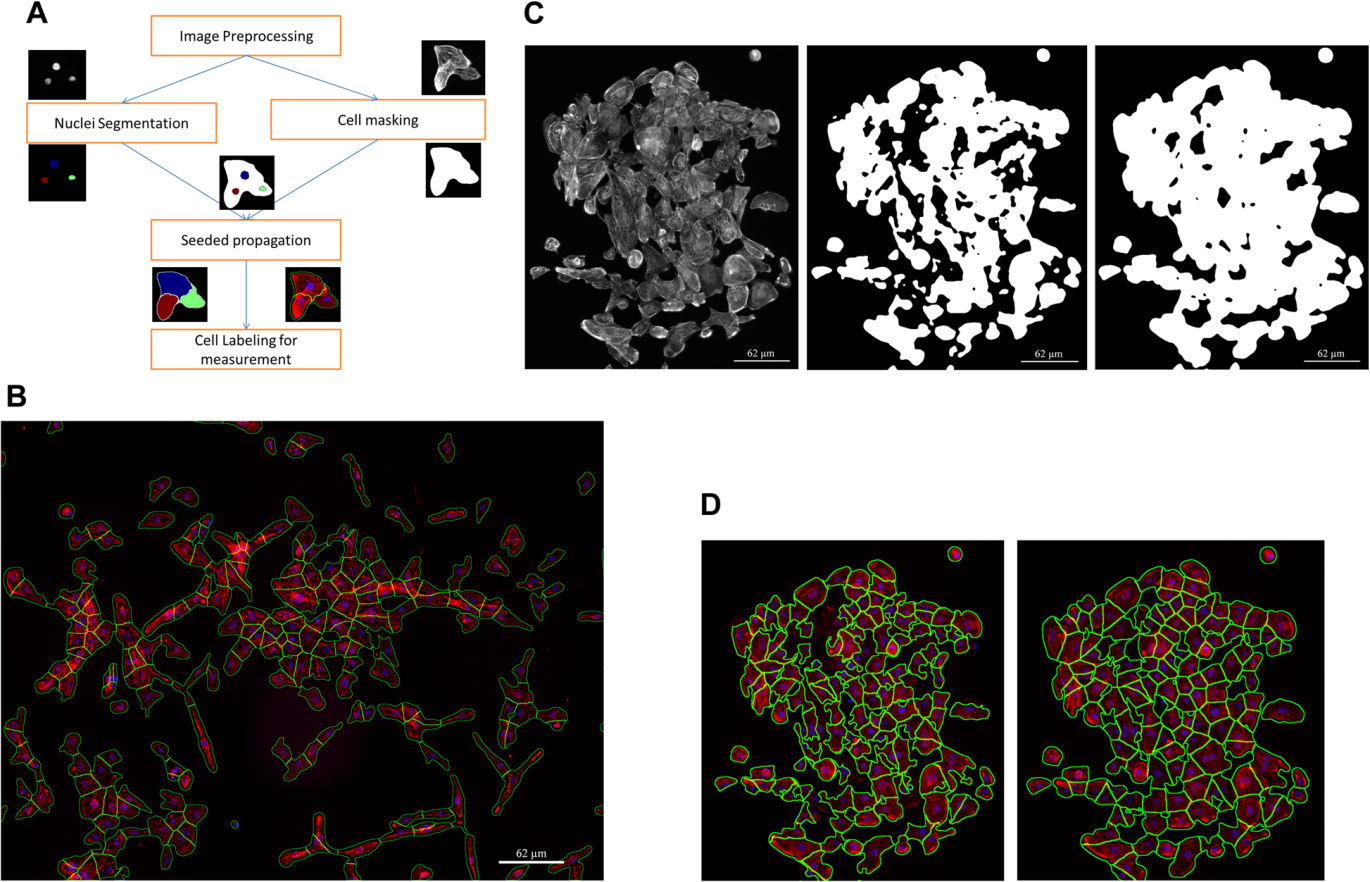
Image analysis pipeline and segmentation results. **a** Image analysis pipeline for segmentation and quantification of the individual cardiomyocytes from the image with *α*-actinin and DAPI staining. **b** A sample of segmentation results (segmentation lines: Green; *α*-actinin signal: Red; DAPI signal: blue). **c** Comparison of cell masking methods on a typical cluster of cells. Left, original image; middle, binary mask of Otsu thresholding method; right, binary mask of Fuzzy c-mean clustering method. **d** Comparison of different segmentation methods. The difference of the two methods are the usage of different cell masking method. Left, Propagation result with Otsu cell masking; right, our propagation result with FCM cell masking

An image analysis pipeline was designed as depicted in Fig. 2a, with the specific goal of segmenting the individual cardiomyocytes from the image with *α*-actinin and DAPI staining. In this manner the phenotype quantification can be based on single cells. The individual steps of the pipeline are discussed in detail in the following sections, but, in short, the signal of the image was enhanced beforehand in an image preprocessing step, a nuclei mask was extracted from the DAPI signal and a cell mask was extracted from the *α*-actinin channel using varied thresholding methods. Subsequently, the single nuclei were identified in the nuclei mask using a watershed segmentation based on distance mapping in the threshold binary image. The segmented single nuclei mask was employed as a seed and propagated in the cell mask to find the cell border. The automated image analysis method was developed as a Java plugin in ImageJ software [23].

#### Image preprocessing

In order to accomplish a better segmentation result, a number of standard image preprocessing steps were performed including background subtraction (ImageJ, Subtract Background, 100 for *α*-actinin channel and 50 for DAPI channel), Gaussian smoothing filter (ImageJ, Gaussian Blur, radius 5 for *α*-actinin channel and radius 2 for DAPI channel), median filter (ImageJ, Median, radius 5 for *α*-actinin channel), and contrast enhancement (ImageJ, Enhance Contrast, Saturated pixels: 0.3% for *α*-actinin channel) so as to reduce the noisy background influence and improve the foreground signal. The background noise is considered to consist of photon noise and some aspecific background fluorescence.

#### Image segmentation

There are three main steps for the image segmentation. First, detection of nuclei to provide seed locations for the cell propagation. Second, cell detection to extract the *α*-actinin positive cell region for the cell propagation. Finally, a seeded cell propagation is initiated to find the border of the individual cells. An example of a segmentation result for the entire image is depicted in Fig. 2b.

##### Nuclei detection

We used the Otsu thresholding method [24] to sufficiently segment the blue channel in order to obtain a binary mask from the DAPI stained nuclei. Subsequently, individual nuclei were detected using a watershed segmentation method. There are two classical watershed segmentation methods: one is based on the distance transformation from the binary mask [25]; the other one is based on the grayscale from the original image [26]. If the nuclei are sparsely distributed in the image, the watershed method based on binary mask will be sufficient for single nuclei identification. If a relatively large amount of nuclei are clustered together, the watershed method based on the grayscale image might result in a better performance. On the other hand, if the signal in the single nuclei is not evenly distributed, the grayscale watershed method might "overcut" the nuclei. This can be complicated further by the fact that cardiomyocytes can have more than one nucleus per cell, although for hiPSC-CMs, the likelihood is not that high, since less than 20% of embryonic stem cell-derived cardiomyocytes are multinucleated [19]. In our case, the cells were cultured in monolayers, which minimized the number of clustered nuclei.

In order to evaluate the performance of the two watershed segmentation methods in our nuclei image dataset, we calculated the percentage of correctly segmented nuclei number for both methods. A reference data set for this analysis was obtained by an evaluation that was carried out by two independent researchers, manually assigning nuclei in 15 randomly selected images (512x512 pixels). The images were exposed to the same preprocessing pipeline before the watershed segmentation method. The result, as shown in Additional file 1: Table S1, demonstrates that 97.8% of the nuclei are correctly segmented using the watershed method based on binary mask whereas 82% of the nuclei are correctly segmented by the watershed method based on the grayscale. From the experiment, we observed that all of the incorrectly segmented nuclei from the latter method, being 18%, suffered from overcutting.

From our pilot experiments we decided to use the watershed segmentation based on the distance transformation so as to prevent the overcutting from the grayscale based watershed method. In order to include bi-nucleated cardiomyocytes but not to overdo the process, only closely located nuclei were merged by applying a consecutive one pixel dilation and erosion in the binary mask. The merged nuclei were treated as one seed for the follow up steps. Finally, the nuclei mask was relabeled as our seed for the propagation.

##### Cell detection

Before we were able to use the single nuclei mask in a seed-propagation step to identify individual cells, we first needed to identify the positive signal in the red-channel, i.e. representing *α*-actinin fluorescence. First of all, an intensity thresholding method was required to separate cell signal from the background. However, *α*-actinin stained hiPSC-CMs displayed unevenly expressed signals in and between cells with a high percentage of weak signals in the image and as a result the *α*-actinin channel did not show a bi-modal histogram but instead a uni-modal histogram with a long tail of high intensity signals. Therefore, for this type of signal Otsu’s thresholding method cannot be applied as it excludes too much signal, as shown in Fig. 2d.

It has been reported that the Otsu thresholding method shows less tolerance with intensity variance [27, 28]. Instead, a machine learning based segmentation method, known as Fuzzy C-Means Clustering Algorithm (FCM) [29] incorporated more tolerance to the intensity variation and therefore it was selected for the thresholding of this channel in our image dataset.

The FCM Algorithm assigns a degree of “belonging to foreground” for each pixel and sets the cut-off between foreground and background based on the minimization of intra-cluster variance. This method can successfully threshold an image with mixed strong and weak signals as shown in Fig. 2c. However, due to the high resolution of our images (4700x3600 pixels per image), it took relatively long (10 minutes in a system with 3.40 GHz processing speed and 16 GB RAM) to extract the cell signal from a single image.

In order to solve the problems with computational load, several improved versions based on FCM have been reported [30]. An accelerated version of the FCM Algorithm called EnFCM [31] was chosen to speed up the segmentation step. EnFCM treats each gray value from the histogram as a clustering candidate rather than each pixel from the image. Therefore, we minimized an energy function, which is expressed (equation 1) in an objective function, as follows:

$$ J_{EnFCM} = \sum_{i=1}^{C}\sum_{l=1}^{q}h_{l}u_{il}^{m}(l-C_{i})^{2}\;\;\;m> 1. $$

where *C* represents the number of clusters, *q* represents the number of gray levels, *h*_*l*_ is the number of pixels whose gray value equals to *l*, *m* is the fuzzyfication parameter which is a real number greater than 1, $u_{il}^{m}$ is the degree of membership of gray level *l*, *C*_*i*_ is the center of the cluster. The iterative optimization of the objective function is carried out by updating the membership *u*_*il*_ and the cluster centers *C*_*i*_:

$$u_{il} = \frac{(C_{i}-l)^{-2/(m-1)}}{\sum_{j=1}^{C}(C_{j}-l)^{-2/(m-1)}} \;\;\;\forall i= 1...c,\;\; \forall l = 1....q, $$

$$C_{i} = \frac{\sum_{l=1}^{q}h_{l}u_{il}^{m}l}{\sum_{l=1}^{q}h_{l}u_{il}^{m}}\;\;\;\forall i = 1...c. $$

In this way, the computation time is drastically reduced by using the histogram instead of using individual pixels; i.e. 2-3 seconds in the system with 3.40 GHz processing speed and 16 GB RAM. This was adequate for our image data processing.

##### Seed propagation

After obtaining the cell mask and the individual nuclei mask, individual cells were identified using a nuclear propagation approach [32]. This approach uses nuclei as the initial seed and propagates the region until it reaches the cell border by comparing both intensity and distance of the neighborhood pixels. The approach also includes a regularization factor to provide reasonable behavior in the case that the image data does not contain strong enough edges, i.e. intensity changes, between two seed regions. In our study, the regularization factor is set to 1, which means that the intensity difference and distance of the neighborhood pixels have the same impact on the propagation. Since *α*-actinin stains the sarcomere structure of the cardiomyocyte, it cannot provide a strong edge signal when the cells are strongly clustered together. This propagation method can, therefore, assist to construct the cell border both in spread out cardiomyocytes (∼500 cells/ *mm*^2^) as well as highly clustered cardiomyocytes (∼2000 cells/ *mm*^2^).

### Segmentation performance assessment

First, a qualitative performance assessment was set out by comparing our segmentation method with a representative seeded segmentation method [1] in our image dataset. We compared the performance by observing the segmentation results as shown in Fig. 2d.

Furthermore, a quantitative performance assessment was conducted by comparing the results of the developed automated image analysis pipeline with manual segmentation results. First, we cropped 15 individual images (512x512 pixels) including 232 cells in total from the original image dataset with varied treatment conditions. Second, two scientists with and without the knowledge of our cardiotoxicity study were asked to independently segment the individual cells from the images. Third, we used the traditional F-score to assess the accuracy of the segmentation methods [33]. The F-score takes is based on a calculation that takes into account both recall and precision. Recall (also known as sensitivity) is the proportion of real positive results that are correctly predicted positive. Precision denotes the proportion of predicted positive results that are correctly real positive [34]. The F-score is then measured as follows:

$$F_{score} = 2\cdot \frac{recall \cdot precision}{recall + precision} $$

We computed the F-score for our method and compared it to the two manually segmented results. We also compute the F-score between the two manually segmented results so as to check the degree of variation between two scorers. Finally, as a point of reference, we segmented the sample images using the conventional Otsu-based segmentation method [1] and computed the F-score of this method compared to a manual segmentation.

### Phenotype measurement

In this study, we included a list of phenotype measurements on the level of single cells in order to describe the changes between different experimental treatment conditions. They can be mainly separated into four main categories: (1) basic measurements, such as cell area, perimeter and mean intensity; (2) shape measurements [35], including extension, dispersion, elongation, compactness, long axis and short axis; (3) texture measurements [36], such as standard deviation of the intensity, smoothness, skewness, uniformity and entropy as shown in Table 1; (4) other measurements such as cell number and cell-cell contact [1], which is the percentage of the cell borders shared with other cells. Each treated condition was compared with control (DMSO) condition using Two-sample Kolmogorov-Smirnov test [37]; i.e. P<0.05 was considered as significant.

**Table 1:** Texture measurements

Feature Name	Expression	Description
std	$f_{1} = \sqrt {\sum _{i}(i-mean)^{2}H(i)} $	The standard deviation of intensity from all the pixels in a region.
Smoothness	$f_{2} = 1 - \frac {1}{(1+f_{1}^{2})} $	The relative smoothness of the intensity in a region of constant intensity in a region. It is 0 for a region of constant intensity and 1 for a region with large excursion in the values of its intensity levels.
Skewness	$f_{3} = \sum _{i}(i-mean)^{3}H(i) $	The order moment about the mean. The departure from symmetry about the mean intensity. It is 0 for symmetric histograms, positive for histograms skewed to the right and negative for histograms skewed to the left.
Uniformity	$ f_{4} = \sum _{i}H^{2}(i) $	The sum of squared elements in Histogram. It reaches maximum when all intensity levels are equal and decreases from there.
Entropy	$ f_{5} = -\sum _{i}H(i)log_{2}H(i) $	The statistical measure of randomness.
i represents the intensity value. *H*(*i*) is the histogram of intensity.		
*mean* symbolizes the average intensity.		

## Results

### Cell masking performance assessment

We evaluated our high-throughput image analysis pipeline by applying it on a dataset of 120 images of hiPSC-CMs (4700x3600 pixels per image), either cultured in control conditions or treated with anticancer drugs with five replicates for each condition. We did the experiment on two different batches of cells from Pluriomics BV and two individual plates in total. We performed dose-response studies using anticancer drugs doxorubicin (a classical anthracycline antibiotic) and crizotinib (a novel tyrosine kinase inhibitor).

The biggest challenge in our study is to perform proper cell masking for the *α*-actinin-stained hiPSC-CMs (Fig. 2c). We compared the performance of a conventional Otsu-based segmentation method, which has been used successfully for segmentation of primary cardiomyocytes in an earlier study [1], with our own method.

We applied both our method and the Otsu-based segmenation method on our data set. The cell masking results are shown in Fig. 2c. The final single cell segmentation results are shown in Fig. 2d. Our method is able to identify both strong and weak signals from the red- channel (*α*-actinin) using the EnFCM thresholding method (Fig. 2c(iii), d(ii)), whereas in the conventional method much of the weak signal is excluded (Fig. 2c(ii), d(i)).

**Fig. 3:**
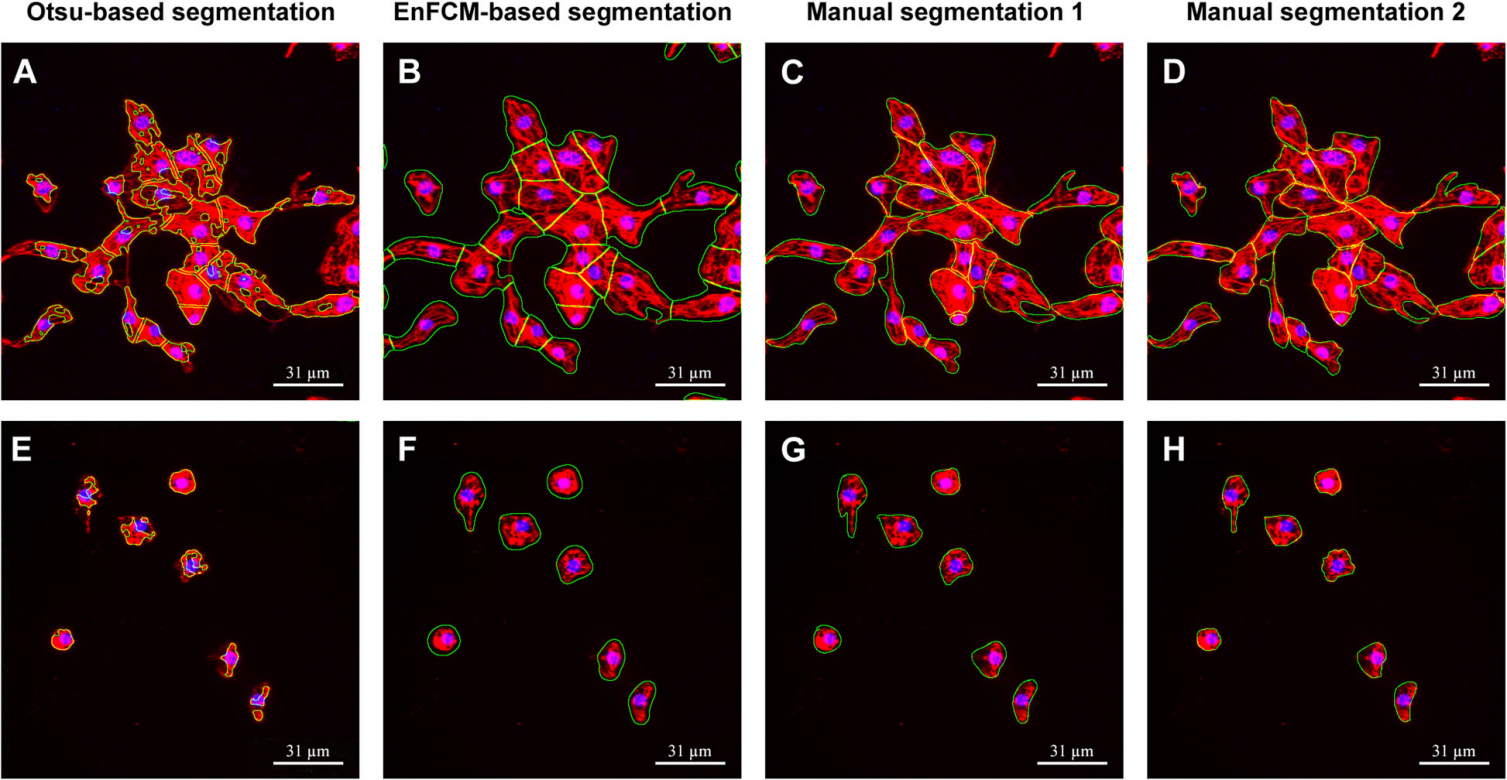
Examples of automated and manual segmentation results. **a**-**d** are images from control conditions and **e**-**h** are from treated conditions with 3 *μ*M crizotinib. **a** and **e** are derived from conventional Otsu-based segmentation. **b** and **f** are derived from our method. **c** and **g** are derived from the first researcher by manual segmentation. (D) and (H) are derived from the second researcher by manual segmentation

To quantify the performance of the segmentation methods, two researchers were asked to manually segment 232 cells from 15 randomly selected images from our sample set with varied treatment conditions as shown in Additional file 1: Table S2. A typical example of these results from the two manual segmentations is shown in comparison to the obtained results of the automated segmentation by our methods and the Otsu-based segmentation method (Fig. 3). Researchers are able to identify individual cells easily when the cells are spread out (Fig. 3e-h). In contrast, it is more difficult for the researchers to precisely identify the cell border in aggregated cells (Fig. 3a-d), especially because the *α*-actinin signal is uneven and cells are very close to each other. Therefore, variation exists between the two sets of manual segmentation results, leading to an overall F-score of 89.88% between the two researchers.

The results of F-score analysis of all cell masking methods are summarized in Table 2. When using the two sets of manual segmentations as a baseline, our method has a higher recall score (91.97%, 93.84%, resp.), than the conventional method (55.29%, 61.23%, resp.). The very low recall score of the conventional method is probably caused as a result of the Otsu thresholding, which fails to select all *α*-actinin signal and only picks up strong *α*-actinin signal from the image. This exclusive selection of high-intensity signal also explains the extremely high precision of the conventional method (97.28%, 97.25%, resp.) when compared to our method (84.28% and 78.49%, resp.). The relatively low precision score of our method is partially caused by the high radius used in the Gaussian filter in the pre-processing stage (5 pixels) in order to smooth the *α*-actinin signal. It brings more neighboring pixels (∼4 pixels) around the *α*-actinin signal into foreground. This is clearly visible in Fig. 3f, but it does not significantly affect the morphological descriptors for single cells as illustrated in the next section.

**Table 2:** F-score analysis for the automated and manual segmentation results

		Automated vs Manual 1			Automated vs Manual 2		
		Precision	Recall	F-Score	Precision	Recall	F-Score
Gregory’s method	Mean	0.9728	0.5529	0.6945	0.9725	0.6123	0.7432
	SEM	0.0055	0.0361	0.0296	0.0047	0.0342	0.0263
Our method	Mean	0.8428	0.9197	0.8775	0.7849	0.9384	0.8533
	SEM	0.0122	0.0130	0.0054	0.0116	0.0102	0.0055

As indicated above, the F-score between the two manual scorers is 89.88%, which is quite close to the average of F-score between our method and the two manual scorers, which is 86.58% (Table 2). This means that our algorithm performs well and is close to the typical variation that is observed in manual scoring. In summary, the overall segmentation performance F-score improved significantly from 71.89% using the conventional method to 86.58% using our method, which is comparable or even higher than the performance of previously reported cell segmentation methods [1, 33]. It should be noted that our method is applied on human cardiomyocytes and uses very large, high resolution, images as input.

### Single-Cell segmentation performance assessment

We also quantified how various automated segmentation methods affect the morphological descriptors for single cells (as described in Supplementary Methods 1). The averages of the distance between the various automated methods and the masks from manual researchers are shown in Additional file 1: Table S3. The Student t-test results in Additional file 1: Table S4 show that there are four single-cell features (area, extension, dispersion and compactness) for which the results of the Otsu-based segmentation method and our method differ significantly. Together with Additional file 1: Table S3, we can see that for three of the four features (area, dispersion and compactness) the result of our method is significantly closer to the manual mask than the Otsu-based segmentation method. For one feature (extension) the conventional method has a significantly closer to the manual masks than our method. This is due to the nature of the extension feature, which has no internal normalization and therefore is very sensitive to slight over segmentation. We therefore conclude that a possible slight overestimation of cell borders in our method does not significantly bias results on different parameters, such as cell number or cell shape and the phenotype changes can still be properly detected. The effect of a partial selection as a result of Otsu thresholding in the conventional method, however, hampers the reliable quantification of morphological descriptors.

### Cell number

**Fig. 4:**
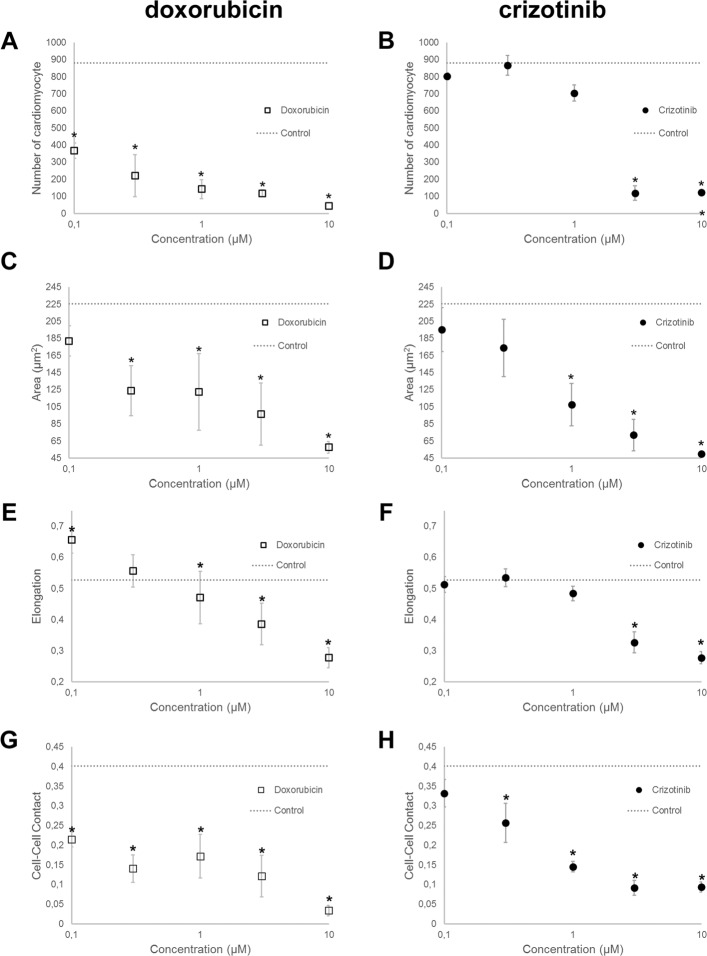
Representative results of phenotype measurements on single cell level. **a** The effects of doxorubicin treatment on cell viability (depicted as number of cardiomyocyte). **b** The effects of crizotinib treatment on cell viability (depicted as number of cardiomyocyte). **c** The effects of doxorubicin treatment on cell area. **d** The effects of crizotinib treatment on cell area. **e** The effects of doxorubicin treatment on cell shape (Elongation). **f** The effects of crizotinib treatment on cell shape (Elongation). **g** The effects of doxorubicin treatment on cell-cell contact. **h** The effects of crizotinib treatment on cell-cell contact. Cells treated with dimethylsulfoxide (DMSO 4.23 mM) is considered as control. In general, data are represented as mean ±s.e.m. *p <0.05 by Two-sample Kolmogorov-Smirnov test. N-number is 5

Two representative anti-cancer drugs with known clinical cardiotoxicity outcomes were tested for cardiotoxicity effects on cardiomyocytes. As our primary testing, we varied the concentration of the drugs at the range between 0.1 *μ*M and 10 *μ*M, which is within the range of clinical use. The total number of cardiomyocytes in the image was used to quantify cell viability. As shown in Fig. 4a-b, both drugs reduced cell viability with increasing drug concentrations. (DMSO treated) condition, doxorubicin reduced the cell number drastically (41.7% of the number of cells compared to DMSO treated control) at the lowest concentration (0.1 *μ*M) and kept on reducing with increasing concentrations of 0.3 *μ*M (25.1%), 1 *μ*M (16.2%), 3 *μ*M (13.3%) and 10 *μ*M (5.0%). Treatment with crizotinib showed a gradual decrease of cell number at the lower concentrations, indicated by 91.1%, 98.4% and 79.9% for the concentration range of 0.1 *μ*M, 0.3 *μ*M, 1 *μ*M. There was a drastic decrease in the cell number at the concentration of 3 *μ*M (13.5%), which remained approximately at the same level at the highest concentration of 10 *μ*M (13.8%). In conclusion, these results showed that both doxorubicin and crizotinib led to cardiomyocyte loss at concentrations less than 10 *μ*M.

### Morphology

In order to detect the morphological changes that could be related to cardiotoxicity, a list of shape features was calculated and four of them are presented in Fig. 4c-f and Additional file 1: Figure S1A-D. These are area, perimeter, elongation and compactness. Morphological changes by these shape descriptors may indicate changes of cell viability and alteration of specific signalling pathways related to cardiotoxicity. Cell size measured by area and perimeter decreases with increasing dose concentrations for both doxorubicin and crizotinib. This is similar to the trend seen in cell density.

Elongation is a measure of how much the shape must be compressed along its long axis in order to minimize its extension [35]. The compactness of an object, also known as circularity, is defined as a function of the perimeter P and the area A, 4A/P2. The circularity of a circle is 1, and less than one for an irregular shape. As compared to control (DMSO treated) cells, doxorubicin-treated cells demonstrated an elongated spindle-like cellular morphology at the lower concentration (0.1 *μ*M and 0.3 *μ*M). At the higher concentrations of doxorubicin and crizotinib (3 *μ*M and 10 *μ*M) cells became smaller and more circular, which would typically be a sign of cells detaching and dying.

### Cell-cell contacts

According to our observations, healthy cardiomyocytes tend to contact to each other and form a tight network [38]. When the cells lose their viability, they start to detach and die. As a result, cell-cell contacts could also be a valid measurement for the cell viability. We observed a significant reduction in cell-cell contacts after adding the drugs when comparing to the control (DMSO treated) condition (Fig. 4g-h).

### Texture

Compared to control (DMSO treated) cells, crizotinib-treated cells have condensed nuclei which may be the cause of drug-induced cardiotoxicity [18]. Therefore, we checked the texture changes in the DAPI channel with different treatments. In Additional file 1: Figure S1E-F the coefficient of variation (CV value) of nuclei intensity is presented. The coefficient of variation indicates the intensity fluctuations in the nuclei region and is defined as the standard deviation of intensity divided by the mean intensity value. The cells treated with crizotinib showed an increasing CV value compared to control cells as well as the cells treated with doxorubicin. The nucleus with condensed chromatin could imply that the cardiomyocyte is in the apoptosis stage.

## Discussion

We developed a fully automated image analysis system that reliably segments cells even with heterogeneous signals and provides single cell information on cardiotoxicity. The segmentation pipeline as included in our image analysis system can correctly detect wide ranges of *α*-actinin signals, thereby allowing the analysis of a broad range of cardiomyocytes, including immature hiPSC-CMs. Following treatment with two anticancer drugs i.e. doxorubicin and crizotinib, we observed loss of cardiomyocytes at increasing drug concentrations. In addition, we observed differences in morphological and texture features, which may provide a better insight on different aspects of drug-induced cardiomyocyte toxicity from different classes of anticancer, or other, drugs. Our current setup is not designed for monitoring live cells. Observing live cells would add extra value on drug dose effects, however, it has not being the purpose of our study.

A large high-content screening with automated image analysis system is necessary to unravel drug-induced cardiotoxicity. Current high-content analysis is mainly based on live calcium signaling in cardiomyocytes to analyse calcium transients using a calcium indicator [39]. We present a method that can analyse phenotype changes related to cardiotoxicity based on images of immunofluorescence. We compared our method to an established mouse cardiomyocyte image analysis system embedded in CellProfiler: the Otsu-based segmentation method. We replaced the more conventional Otsu thresholding method to EnFCM clustering algorithm in order to properly segment cells based on unevenly distributed *α*-actinin signals in hiPSC-CMs. With our segmentation method we improved the F-score from 71.89% using Otsu-based segmentation to 86.58% with our EnFCM-based segmentation.

Phenotypical changes are crucial for the analysis of cardiotoxicity. The difference of phenotypical changes between drug treatments enlightens the identification of specific signalling pathways related to cardiotoxicity for further exploration. We have been able to quantify a list of phenotype measurements including shape, texture and other related features so as to form a unique phenotype matrix for different drugs.

Our image analysis system is essentially helpful for drug testing on cardiotoxicity since it is required for all drugs before entering clinical trials and ultimately the market. Furthermore, the Food and Drug Administration (FDA) is in favour of performing these test in human stem cell based assays [40]. In addition to drug-induced cardiotoxicity, differences in cardiomyocyte morphology and texture related to sarcomeric organization are also strongly associated with diseased or dysfunctional cardiomyocytes. Therefore, hiPSC-CMs derived from patients suffering from cardiac disease can be evaluated using our improved analysis system, which will be helpful to understand the underlying mechanisms and to identify doses of drugs or compounds that can rescue the cardiomyocyte disease phenotype. For future work, we will explore deep learning models, especially Convolutional Neural Network (CNN) based segmentation methods such as U-Net [41] and Mask-RCNN [42]. These are two popular segmentation methods and seem to be outstanding models for nuclei segmentation challenges. In addition, these models are even joint to further improve the performance of Nuclei segmentation[43]. We intend to evaluate the performance of these two models for the segmentation of highly clustered hiPSC-CMs. As a starting point, our validated segmentation method can be successfully used as input to these deep learning models. In our study, we have shown the success rate of our method and the application gives us around 43870 cardiomyocytes which can be used as training data for a U-net or Mask-RCNN model. We need to make the comparison to see which setup has a better performance. To the best of our knowledge, this number of training samples seems to be sufficient to train the deep learning models and assess their performance. Subsequently, in the future, we hope we can include a deep net in our standardized pipeline. Moreover, our research will include an assessment for distributed computing so as to balance the computational load between GPU and CPU.

## Conclusions

Our method has shown to be particularly efficient in the processing of large images of hiPSC-CMs. We showed the potential of our system for determining cardiotoxicity based on phenotypical changes in hiPSC-CMs. This high-throughput assay could be further enhanced by combining with other high-throughput assays using functional and biochemical parameters, such as cardiomyocyte contractility, electrophysiology, calcium signalling and mitochondrial activity [44]. By combining such high-throughput assays we can collect a profound set of data to describe the phenotype variations of hiPSC-CMs in response to drugs, cardiac disease, or the combination thereof. In summary, our image analysis system is an automated and accurate solution for the evaluation of drug-induced cardiotoxicity in hiPSC-CMs.

## Supplementary information

**Additional file 1** This additional file provides one supplementary figure, four supplementary tables and extra explanation of method.

## Data Availability

The datasets used and/or analysed during the current study are available from the corresponding author on reasonable request.

## Abbreviations

hiPSC: Human somatic cells induced pluripotent stem cells
hiPSC-CMs: Human induced pluripotent stem cell-derived cardiomyocytes
DMSO: Dimethylsulfoxide
DPBS: Dulbecco’s phosphate-buffered saline
DAPI: 4’,6-diamidino-2-phenylindole
FCM: Fuzzy C-Means Clustering Algorithm
EnFCM: Enhanced Fuzzy C-Means Clustering Algorithm
CV: Coefficient of variation
CNN: Convolutional Neural Network
