# Supplementary material for: Automated image analysis system for studying cardiotoxicity in human pluripotent stem cell-Derived cardiomyocytes
Source: BMC Bioinformatics. 2020 May 14;21:187. doi: 10.1186/s12859-020-3466-1 (PMC7222481; doi:10.1186/s12859-020-3466-1)
Supplement: Supplementary file 1 — Additional file 1 This additional file provides one supplementary figure, four supplementary tables and extra explanation of method. [file 12859_2020_3466_MOESM1_ESM.zip › 12859_2020_3466_MOESM1_ESM/bmc_supplementary_version2.pdf]

METHODOLOGY ARTICLE

# Supplementary Material for Automated Image Analysis System for Studying Cardiotoxicity in Human Pluripotent Stem Cell-Derived Cardiomyocytes

Lu Cao[??], Andries van der Meer[??], Fons J. Verbeek[??] and Robert Passier[??,??*]

[*]Correspondence:
robert.passier@utwente.nl
[??]Dept of Applied Stem Cell Technologies, MIRA Institute, University of Twente, Drienerlolaan 5, 7522 NB Enschede, The Netherlands
Full list of author information is available at the end of the article

**Abstract**
In this supplement, we provide extra result figure and tables. We further explain the method on how the distance of morphological descriptors between different methods are calculated.

## Supplementary Methods.

We quantified 10 morphological descriptors from manually delineated masks and automated segmentation methods (Gregory and our method). We calculated the mean of each morphological descriptors at each sample images (15 images in total) based on single cell level and derived a 15x10 matrix of morphological descriptors for each methods (15 images x 10 features). We then calculated the Euclidean distance of morphological descriptors between automated methods and the manual masks as shown in Equation 1.

$$D = \|A_i - G_j\|, i = 1, 2; j = 1, 2 \tag{1}$$

$A_i$ is a 15x10 matrix representing the morphological descriptors of an automated methods. $G_j$ is a 15x10 matrix representing the morphological descriptors of one of the manual delineated masks. D is a 15x10 matrix representing the distance of morphological descriptors between automated methods and manual masks. The averages of the distance in 15 images are shown in Table 2. In order to see if the distance of morphological descriptors between Gregory's method versus manual masks is significant higher or lower than the distance between our method versus manual masks. We did a paired Student's t-test as shown in Equation 2 for the distance of morphological descriptors between Gregory's method versus mean of manual masks and our method versus mean of manual masks as shown in Table 4.

$$\begin{aligned} H &= ttest(D_{1,mean}, D_{2,mean}) \\ D_{1,mean} &= \|A_1 - G_{mean}\|, \\ D_{2,mean} &= \|A_2 - G_{mean}\|, \\ G_{mean} &= \frac{G_1 + G_2}{2} \end{aligned} \tag{2}$$

## Supplementary Tables.

**Table 1 Results of the two watershed methods.**

| Image# | Grayscale Watershed | | Binary Watershed | | Ground truth |
|---|---|---|---|---|---|
| | correct nucleus | over-cut nucleus | correct nucleus | undercut nucleus | nucleus |
| 01 | 9 | 0 | 9 | 0 | 9 |
| 02 | 13 | 4 | 17 | 0 | 17 |
| 03 | 13 | 1 | 14 | 0 | 14 |
| 04 | 13 | 3 | 16 | 0 | 16 |
| 05 | 9 | 1 | 10 | 0 | 10 |
| 06 | 7 | 0 | 7 | 0 | 7 |
| 07 | 12 | 3 | 15 | 0 | 15 |
| 08 | 7 | 0 | 7 | 0 | 7 |
| 09 | 17 | 4 | 21 | 0 | 21 |
| 10 | 16 | 4 | 16 | 4 | 20 |
| 11 | 12 | 7 | 19 | 0 | 19 |
| 12 | 18 | 2 | 18 | 2 | 20 |
| 13 | 37 | 12 | 49 | 0 | 49 |
| 14 | 15 | 5 | 20 | 0 | 20 |
| 15 | 21 | 2 | 23 | 0 | 23 |
| Total | 219 | 48 | 261 | 6 | 267 |

**Table 2 Source of the 15 sample images.**

| Image number | condition |
|---|---|
| 01 | 10 µM Crizotinib |
| 02 | DMSO |
| 03 | DMSO |
| 04 | Untreated |
| 05 | Untreated |
| 06 | DMSO |
| 07 | 3 µM Crizotinib |
| 08 | 3 µM Crizotinib |
| 09 | 3 µM Doxorubicin |
| 10 | 1 µM Crizotinib |
| 11 | 1 µM Doxorubicin |
| 12 | 0.3 µM Crizotinib |
| 13 | 0.3 µM Crizotinib |
| 14 | 0.3 µM Doxorubicin |
| 15 | DMSO |

**Table 3 Distances of morphological descriptors between automated methods and manual masks.**

| feature | GT1 vs Greg. | GT1 vs Ourmed | GT2 vs Greg. | GT2 vs Ourmed |
|---|---|---|---|---|
| area | 844.5085 | 376.6746 | 772.9781 | 473.3616 |
| perimeter | 39.8387 | 22.37887 | 30.50528 | 41.38502 |
| extension | 0.173539 | 0.27932 | 0.170465 | 0.304434 |
| dispersion | 0.134496 | 0.052642 | 0.106277 | 0.086671 |
| elongation | 0.165036 | 0.229541 | 0.152465 | 0.218039 |
| compactness | 0.121011 | 0.044702 | 0.093765 | 0.077006 |
| intensity | 22.87597 | 12.14343 | 17.92873 | 17.09068 |
| int_std | 2.683631 | 3.248024 | 2.144146 | 3.661939 |
| uniformity | 0.00432 | 0.006291 | 0.003638 | 0.00677 |
| entropy | 0.141561 | 0.135514 | 0.126798 | 0.145406 |

**Table 4 Distances of morphological descriptors between automated methods and manual masks.**

| feature | area | perimeter | extension | dispersion | elongation |
|---|---|---|---|---|---|
| h-value | 1 | 0 | 1 | 1 | 0 |
| p-value | 0.0022 | 0.7718 | 0.0395 | 0.0328 | 0.0773 |
| feature | compactness | intensity | int_std | uniformity | entropy |
| h-value | 1 | 1 | 0 | 0 | 0 |
| p-value | 0.0231 | 0.0292 | 0.1907 | 0.1848 | 0.9376 |

## Supplementary Figures.

**Figure 1 Results of phenotype measurements on single cell level.** (A) The effects of doxorubicin treatment on cell perimeter. (B) The effects of crizotinib treatment on cell perimeter. (C) The effects of doxorubicin treatment on cell shape (Compactness). (D) The effects of crizotinib treatment on cell shape (Compactness). (E) The effects of doxorubicin treatment on nuclei texture (Coefficient of Variation). (F) The effects of crizotinib treatment on nuclei texture (Coefficient of Variation). In general, data are represented as mean$\pm$s.e.m. *$p<0.05$ by Two-sample Kolmogorov-Smirnov test. N-number is 5.
