# Supplementary figures and images for: Automated image analysis system for studying cardiotoxicity in human pluripotent stem cell-Derived cardiomyocytes
Source: BMC Bioinformatics. 2020 May 14;21:187. doi: 10.1186/s12859-020-3466-1 (PMC7222481; doi:10.1186/s12859-020-3466-1)

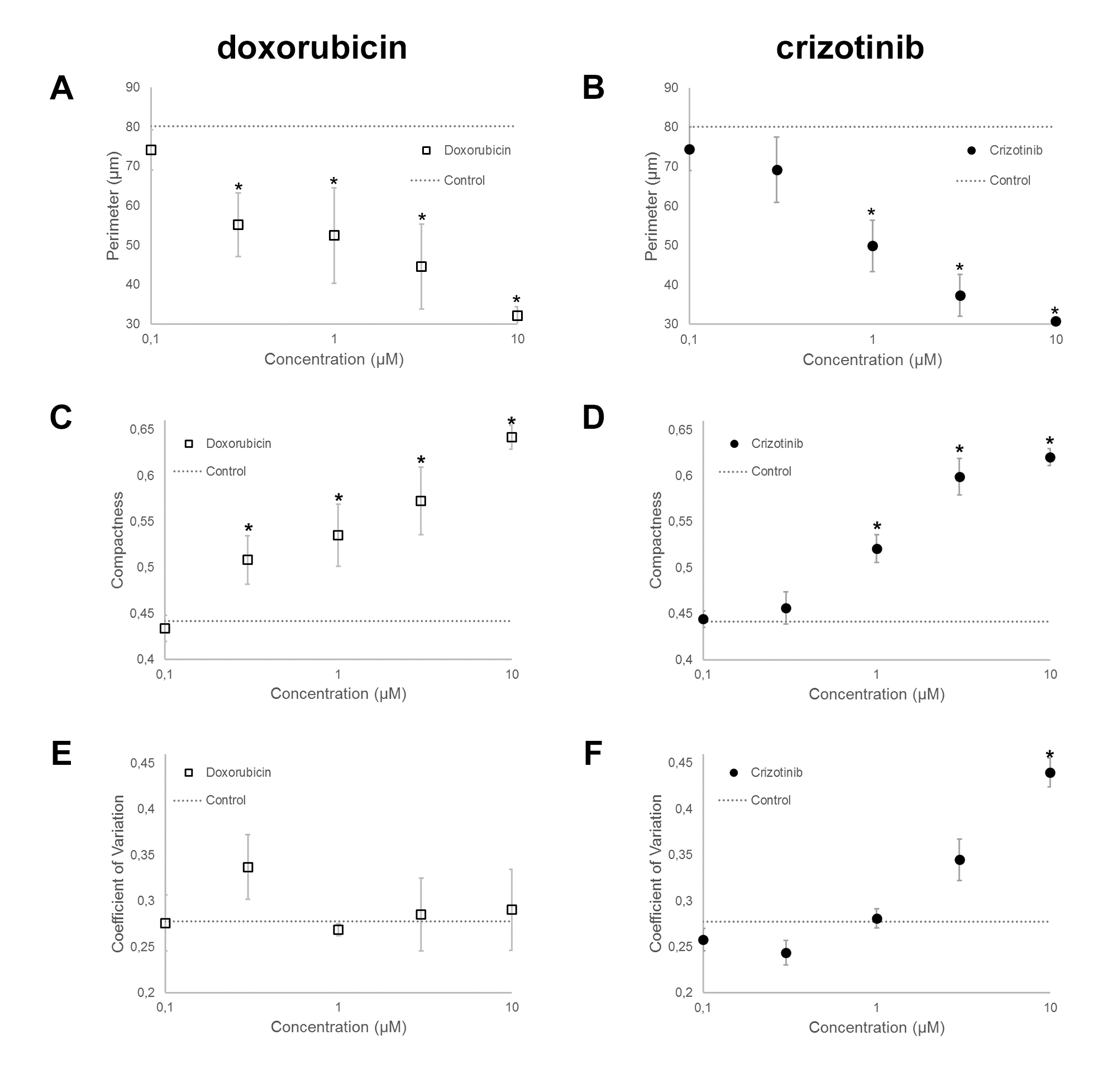

Supplement: Supplementary file 1 — Additional file 1 This additional file provides one supplementary figure, four supplementary tables and extra explanation of method. [file 12859_2020_3466_MOESM1_ESM.zip › 12859_2020_3466_MOESM1_ESM/SupplementaryFigure1.tif]
